# Novel pathogenic variant in *ARMC4* identified by whole exome sequencing in a Turkish family with primary ciliary dyskinesia

**DOI:** 10.3389/fmolb.2026.1815627

**Published:** 2026-06-02

**Authors:** Elif Arik Sever, Rim Hjeij, Pınar Ata, Ela Erdem Eralp, Emine Atag, Heymut Omran, Bulent Taner Karadaǧ, Osman Ugur Sezerman

**Affiliations:** 1 Department of Biostatistics and Bioinformatics, Graduate School of Health Sciences, Acibadem Mehmet Ali Aydinlar University, Istanbul, Türkiye; 2 Department of Medical Biology and Genetics, School of Medicine, Okan University, Istanbul, Türkiye; 3 Department of General Pediatrics, University Children’s Hospital Muenster, Muenster, Germany; 4 Department of Medical Genetics, School of Medicine, Marmara University, Istanbul, Türkiye; 5 Department of Pediatric Pulmonology, School of Medicine, Marmara University, Istanbul, Türkiye; 6 Department of Pediatric Pulmonology, School of Medicine, Maltepe University, Istanbul, Türkiye; 7 Department of Biostatistics and Medical Informatics, School of Medicine, Acibadem Mehmet Ali Aydinlar University, Istanbul, Türkiye

**Keywords:** ARMC4, case report, outer dynein arm, primary ciliary dyskinesia, whole exome sequencing

## Abstract

**Introduction:**

Primary ciliary dyskinesia (PCD) is a rare genetic disorder affecting approximately 1:15,000 to 1:30,000 individuals. It is characterized by upper and lower respiratory diseases, otitis media, congenital heart defects, situs inversus, infertility in males, and hydrocephalus. Whole-exome sequencing (WES) has become an important tool for identifying novel pathogenic variants associated with PCD.

**Methods:**

Trio-based WES was performed in two Turkish siblings with clinically suspected PCD born to healthy consanguineous parents. Candidate variants were validated by Sanger sequencing and functionally assessed using immunofluorescence (IF) analysis of respiratory cilia obtained by nasal brushing biopsy.

**Results:**

A novel homozygous frameshift pathogenic variant in *ARMC4* [c.324dupA; p.Arg109Thrfs*19] was identified in both affected siblings. IF analysis demonstrated absence of *ARMC4* protein in the ciliary axonemes and distal loss of DNAH5, confirming the functional effect of the variant.

**Discussion:**

These findings expand the mutational spectrum of *ARMC4*-associated PCD and highlight the importance of combining genetic and functional analyses for accurate diagnosis and variant interpretation in PCD.

## Introduction

Cilia are hair-like organelles found on most of the eukaryotic cells and they are categorized into two groups: motile and nonmotile (primary). Motile cilia are typically present in large numbers and beat in a coordinated manner on the cell surfaces of organs such as the fallopian tubes, trachea, and brain ventricles (Satir and Christensen, 2017). The functions include cell motility, sensation, signal transduction, embryonic development, cerebrospinal fluid circulation, and fluid movement (Mazor et al., 2011). Structurally, motile cilia contain a microtubule-based axoneme, typically organized in a 9 + 2 arrangement, with dynein arms playing a key role in generating ciliary motion (Basu and Brueckner, 2008).

Primary ciliary dyskinesia (PCD) is a genetic disorder caused by defects in ciliary structure or function, leading to impaired mucociliary clearance. Clinically, PCD is characterized by chronic or recurrent upper and lower respiratory infections, sinusitis, otitis media, and bronchiectasis. Approximately, 50% of affected individuals exhibit laterality defects such as situs inversus. Other associated features include hearing loss, hydrocephalus, male infertility, and subfertility (Kosaki et al., 2004; Kennedy et al., 2007). Diagnosis relies on a combination of clinical and laboratory findings, including the nasal nitric oxide (nNO) measurement (<200 ppb), transmission electron microscopy (TEM), immunofluorescence (IF) analysis, high-speed video microscopy, and genetic testing (Raidt et al., 2022; Shapiro, 2018; Shoemark et al., 2012). Advances in next-generation sequencing (NGS) technologies have facilitated the identification of pathogenic variants, and whole-exome sequencing (WES) is widely used to detect both known and novel variants associated with PCD (Bachmann-Gagescu, 2014).

PCD is an inherited disease with autosomal recessive or, less commonly, X-linked inheritance, affecting approximately one in every 15,000-30,000 individuals. More than 50 genes have been linked with PCD (Wallmeier et al., 2020); however, the genetic cause remains unresolved in a substantial proportion of cases (Shapiro, 2018). Many pathogenic variants occur in genes encoding components of the outer dynein arms (ODAs) and their associated docking complexes, which are essential for proper ciliary motility. Among these, ARMC4 has been identified as a key component of the ODA docking complex, where its disruption leads to defective dynein arm assembly and impaired ciliary function (Hjeij et al., 2013).

In this study, we aimed to determine the genetic cause of PCD in a consanguineous Turkish family using trio-based WES. The family consisted of healthy parents, two affected siblings (FP1 and FP2), and one unaffected sibling (Figure 2A). FP1 and FP2 were born to consanguineous parents. The clinical characteristics of the patients are summarized in Table 1.

**TABLE 1:** Clinical characteristics, genetic findings, and genotype–phenotype correlation in children with primary ciliary dyskinesia (PCD).

Patient	Patient FP1	Patient FP2
Family origin	TR	TR
Sex	Female	Female
Clinical characteristics	Recurrent respiratory infections, wet cough, situs inversus totalis, chronic sinusitis, bronchiectasis, adenoidectomy, tonsillectomy, ear tube, replacement history	Recurrent respiratory infections, wet cough, situs inversus totalis, chronic sinusitis, bronchiectasis, adenoidectomy, tonsillectomy, ear tube, replacement history
nNO (ppb)	18 ppb	17 ppb
Family consanguinity	Yes	Yes
Zygosity	Homozygous	Homozygous
Affected gene	ARMC4	ARMC4
Base and a.acid change	c.324dupA (p.Arg109Thrfs*19)	c.324dupA (p.Arg109Thrfs*19)
IF findings	ARMC4 absent, distal DNAH5 loss	ARMC4 absent, distal DNAH5 loss
Ciliary defect	ODA defect	ODA defect
PICADAR	10	10
FEV1 (%pred)	91	94
FVC (%pred)	83	78
FEV1/FVC (%)	100	110

TR, Turkish; NO, nasal nitric oxide (ppb); IF, immunofluorescence; ODA, outer dynein arms. Genotype is reported according to HGVS nomenclature. IF staining indicates localization of ARMC4 and DNAH5 proteins. Ciliary defects identified by IF correspond to abnormalities in outer dynein arm (ODA) assembly. Genotype–phenotype correlation is based on concordance between genetic findings and IF results.

## Materials and methods

### WES and filtering

Peripheral blood samples (5 mL) were obtained from the affected individuals and their healthy family members. Genomic DNA was extracted from leukocytes using a Zymo Research Quick-DNA isolation kit. DNA concentrations were measured using an IMPLEN PEARL nanophotometer, and samples concentrations >100 (ng/μL) were used for sequencing. WES was performed using the Illumina Hiseq 2500 platform and the Agilent SureSelect XT_V6 Post cap kit. Sequencing reads were aligned to the human reference genome (UCSC hg38).

### Bioinformatic analysis

**FIGURE 1:**
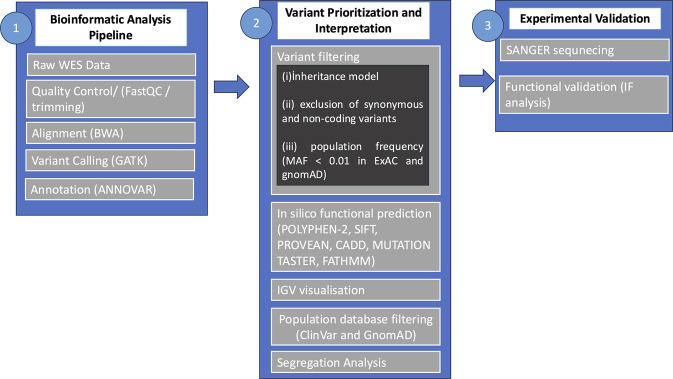
Bioinformatic analysis workflow and variant prioritization strategy. (1) Bioinformatic analysis pipeline: Whole-exome sequencing (WES) data were processed through standard steps including quality control, read alignment, variant calling, and annotation. (2) Variant prioritization and interpretation: Variants were filtered based on inheritance model, population frequency (gnomAD, ExAC), and exclusion of synonymous and non-coding variants. Candidate variants were further evaluated using *in silico* prediction tools (PolyPhen-2, SIFT, PROVEAN, CADD, MutationTaster, FATHMM), visualized using Integrative Genomics Viewer (IGV), and assessed using ClinVar and population databases. Segregation analysis was performed within family. (3) Experimental validation: The identified variant was validated by Sanger sequencing and functionally assessed using immunofluorescence (IF) analysis.

Bioinformatic analysis was performed using a stepwise variant analysis pipeline including read preprocessing, alignment, variant calling, annotation, and filtering. A schematic overview of the workflow is provided in Figure 1. Adapters were removed using Trimmomatic (v 0.36) (Bolger et al., 2014). TruSeq3-PE-2 sequences were used as adapter sequences. Low divergent trimmed reads were aligned to the human hg38 reference genome using the Burrows-Wheel Aligner (BWA mem v0.7.12). PICARD (V1.141) was used to mark duplicate reads (Li and Durbin, 2009; Picard, 2025). Post-alignment processing steps, including indel realignment, base quality score recalibration, variant calling, joint genotyping, and variant quality score recalibration, were performed using GATK (v4.0)^15^. Variants were annotated using ANNOVAR (Wang et al., 2010).

### Variant filtration

Exome variant analyses were performed by filtering the entire variant list according to the following criteria.

- Consistency with autosomal or X-linked recessive inheritance models,
- Exclusion of synonymous and non-coding variants (intergenic, intronic, and UTR regions),
- Minor allele frequency (MAF) <0.01 in ExAC and gnomAD databases,
- Confirmation by Integrative Genomics Viewer (IGV), (Figure 1) (James, 2011).
- Removal of potential false positives using runs of homozygosity (ROH) analysis, (Pike, 2017).
- assessment of functional impact using *in silico* tools (MutationTaster (http://mutationtaster.org/), SIFT, PolyPhen-2, PROVEAN, CADD, and FATHMM), supported by ClinVar database (https://www.ncbi.nlm.nih.gov/clinvar/) annotations.

Variants were prioritized if predicted to be pathogenic by at least three of the six *in silico* tools. (Schwarz et al., 2014; Rentzsch et al., 2021; Ng and Henikoff, 2003; Landrum et al., 2018; Adzhubei et al., 2010; Choi et al., 2012; Shihab et al., 2014; Niroula and Vihinen, 2019).

### Mutation validation and cosegregation analysis

Candidate variants identified by WES were validated by Sanger sequencing. Segregation analysis was performed in the parents and available family members.

Variants from the exome data were resequenced by Sanger sequencing using the original primers and manually optimized primers designed using Primer3 software (*ARMC4* Forward: 5′-AGTGTGGAAGAAAGTGGGAAA-3′, Reverse: 5′-AATCACAGACTGGAGCATTAAGA-3′). Amplicons were sequenced with an amplicon length of 306 bp, and resequencing was performed on an ABI 3130x sequencer system using the BigDye 3.1 sequencing kit (both from Applied Biosystems, Carlsbad, CA, USA). Reads generated by resequencing were aligned to genome build hg38 (NCBI build 38).

### If assay

To confirm loss-of-function mutations in *ARMC4*, IF analysis was performed as previously described (Hjeij et al., 2013). The polyclonal rabbit anti-human DNAH5 was reported previously (Hjeij et al., 2013; Hjeij et al., 2014). The monoclonal mouse anti acetylated-α-tubulin (T7451) was obtained from Sigma (Germany). The polyclonal rabbit anti-human ARMC4 antibody (HPA037829) was obtained from Atlas Antibodies (Sweden). Antibody specificity was confirmed by absent protein localization in the axonemes of patients with corresponding loss-of-function mutations. Anti-mouse Alexa Fluor 488 and anti-rabbit Alexa Fluor 546 were used as the secondary antibodies (Molecular Probes, Invitrogen). DNA was stained with Hoechst 33,342 (Sigma). Images were taken with a Zeiss Apotome Axiovert 200 and processed using AxioVision 4.8, ZEN and Adobe Creative Suite four software.

## Results

### Clinical features

In this study, we performed trio-based WES in a consanguineous family consisting of healthy parents and their affected children. The family included two children with PCD and one unaffected child. The affected children, aged8 (FP1) and 17 (FP2) years respectively, were evaluated in detail. The diagnostic approach was performed in accordance with previously established European Respiratory Society (ERS) recommendations (Lucas et al., 2017), which were applicable at the time of the study. Both patients presented with clinical features highly consistent with PCD, including recurrent upper and lower respiratory tract infections, chronic sinusitis, recurrent otitis media, bronchiectasis, and situs inversus totalis. The PICADAR scores for FP1 and FP2 were calculated as 10 (scores >5 strongly suggest PCD), further supporting the clinical suspicion.

Nasal nitric oxide (nNO) levels were markedly reduced in both patients (18 ppb and 17 ppb), measured using a NIOX MINO device, consistent with PCD. High-speed video microscopy (HSVM) was attempted; however, a complete evaluable assessment could not be obtained, and further analysis was not possible due to lack of consent for repeated procedures. Transmission electron microscopy (TEM) was not available at our center at the time of diagnosis. The diagnosis was therefore based on an integrated approach combining clinical findings, reduced nNO levels, supportive HSVM observations, genetic analysis, and functional validation using immunofluorescence. Chest imaging revealed bronchiectasis in both patients. In addition to dextrocardia, both individuals exhibited situs inversus totalis with mirror-image positioning of abdominal organs (liver and spleen). Neonatal respiratory distress syndrome (RDS) was not observed in either patient. Fertility status has not been evaluated. Pulmonary function tests showed near-normal spirometric values in Patient 1 (FEV1 91% predicted, FVC 94% predicted, FEV1/FVC 100%) and mildly reduced lung function in Patient 2 (FEV1 83% predicted, FVC 78% predicted, FEV1/FVC 110%). Bronchodilator reversibility tests were negative in both patients. Both patients are registered in the international PCD registry (pcdregistry.uni-muenster.de), supporting the clinical diagnosis and phenotypic classification (Table 1).

### Genetic analysis

Following WES data analysis, 1,221,835 heterozygous and 1,078,006 homozygous variants passed the depth and quality filtering for the family. After filtering based on population frequency, synonymous variants, and splice site variants, nine candidate variants remained. Functional significance of these variants was assessed using various *in silico* tools (MutationTaster, PROVEAN, SIFT, PolyPhen-2, CADD, FATHMMM). However, most of these tools (PROVEAN, PolyPhen-2, CADD and FATHMM) are primarily designed to predict single amino acid substitutions and are therefore limited in evaluating frameshift variants. After further prioritization, two candidate variants remained (Table-2) located in *ENKD1* and *ARMC4*. The *ARMC4* variant was not present in gnomAD or ClinVar databases, whereas the *ENKD1* variant has been previously reported in gnomAD, but has no known association with PCD (gnomAD Variant ID:16-67663688-T-C).

**TABLE 2:** Functional prediction and conservation analysis of the identified variants. PROVEAN (D: deleterious), SIFT (D: damaging), NA: not available, N: neutral, B: benign. CADD scores > 20 denote that the variant is pathogenic. Higher scores indicate greater pathogenicity of the variant (Niroula and Vihinen, 2019).

Gene	NM	Base change	Amino acid change	Mutation taster	PROVEAN	SIFT	PolyPhen2	CADD	FATHMM
*ARMC4*	001290020	324dupA	R109fs	Disease-causing	NA	D	NA	NA	NA
*ENKD1*	032,140	A712G	N238D	Disease-causing	N	D	B	21.6	D

**FIGURE 2:**
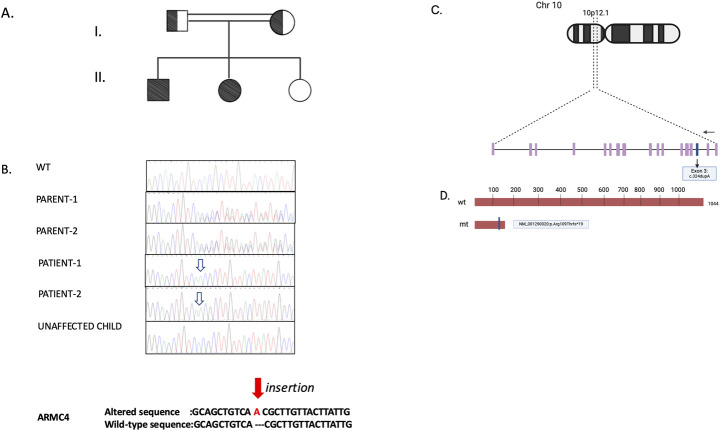
Frameshift variant in *ARMC4* predicted to result in premature truncation identified in two PCD-affected siblings. **(A)** Pedigree of a Turkish family with two PCD-affected children. Roman numerals refer to generations, and individuals within a generation are numbered from left to right. Black circles/squares indicate affected individuals, and white circles/squares indicate unaffected individuals. Half-black/half-white circles/squares denote the heterozygous parents. Two lines between the heterozygous circle and square represent consanguineous marriage. **(B)** Sanger sequencing results of healthy parents with heterozygous variants for *ARMC4* and affected children homozygous variants for *ARMC4*. A duplication of adenine in the altered sequence for the affected children is indicated by arrows. Both healthy parents were heterozygous (Parent-1 and Parent −2), whereas their unaffected child did not carry the variant. **(C)** Schematical chromosome 10 and genomic structure of *ARMC4* (ODAD2-201; NM_018076.5*)*. The position of the adenine duplication is indicated by an arrow and by the vertical blue exon. **(D)** Schematical illustration displays a comparison of the lengths of wild type (wt) and p.Arg109Thrfs*19 mutant (mt) ARMC4 proteins. Adenine duplication leads to an early stop codon by exchanging arginine with threonine at the position 109 illustrated by the vertical blue bar.

**FIGURE 3:**
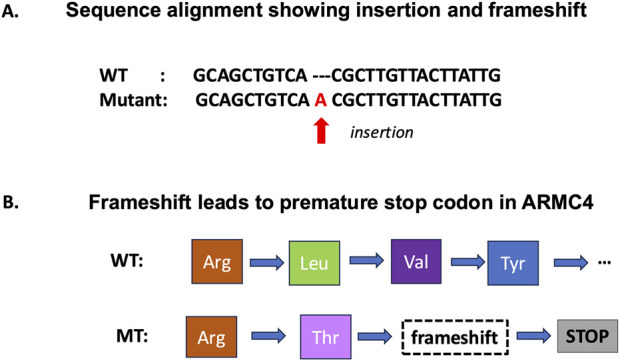
Sequence alignment and predicted effect of the *ARMC4* frameshift variant. **(A)** Alignment of wild-type and mutant nucleotide sequences showing the single nucleotide insertion (highlighted), resulting in a frameshift. **(B)** Schematic representation of the predicted protein-level effect, where the frameshift alters the amino acid sequence and leads to a premature stop codon (p.Arg109Thrfs*19).

Given the inheritance pattern, we focused on variants that were homozygous in the affected individuals and heterozygous in the parents, while absent in the unaffected sibling (Figure 2A). The affected individuals were homozygous for a novel ARMC4 variant consisting of a single nucleotide duplication (NM_001290020:p.Arg109Thrfs*19). This frameshift variant introduces a premature stop codon at amino acid position 127, resulting in a truncated protein (Figures 2C,D). The variant was predicted to be “disease-causing” and “damaging” by MutationTaster and SIFT. It is not reported in population or clinical databases such as ClinVar. Recessive inheritance was confirmed by segregation analysis (Figure 2B). The effect of the insertion on the reading frame and the resulting premature stop codon are illustrated in Figure 3. To further support the pathogenicity of the identified variant, classification was performed according to the American College of Medical Genetics and Genomics and the Association for Molecular Pathology (ACMG/AMP) guidelines. The ARMC4 variant (NM_001290020:c.324dupA; p.Arg109Thrfs*19) was classified as pathogenic based on the following criteria:

PVS1 (Very Strong): The variant is a frameshift duplication leading to a premature stop codon and predicted loss-of-function. Loss-of-function is a well-established disease mechanism in ARMC4-associated PCD.

PM2 (Moderate): The variant is absent from population databases, including gnomAD and ClinVar.

PP1 (Supporting): Co-segregation analysis showed that both affected individuals were homozygous, while the parents were heterozygous carriers.

PP4 (Supporting): The phenotype (bronchiectasis, situs inversus totalis, recurrent respiratory infections, and low nNO levels) is highly specific for PCD.

PS3 (Supporting): Immunofluorescence analysis demonstrated absence of ARMC4 protein and abnormal distal DNAH5 localization, consistent with outer dynein arm dysfunction.

Based on these criteria, the variant was classified as pathogenic according to ACMG/AMP guidelines (Richards et al., 2015).

### If analysis

**FIGURE 4:**
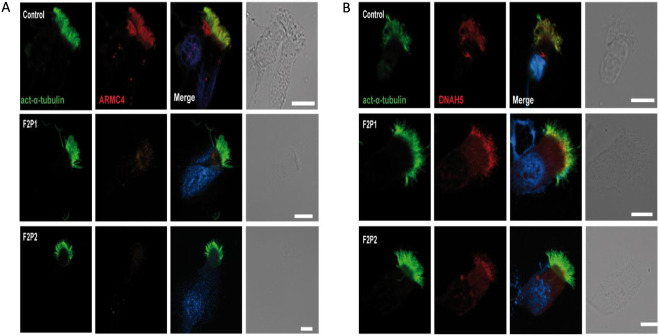
Immunofluorescence analysis showing absence of ARMC4 and distal loss of DNAH5 in affected individuals. **(A,B)** Respiratory epithelial cells from control and affected individuals carrying biallelic *ARMC4* loss-of-function variants [c.324dupA; p.R109fs]. Cells were double-labeled with antibodies directed against acetylated-α-tubulin (green, **(A,B)**, ARMC4 (red, **(A)**, and DNAH5 (red, **(B)**. Nuclei were stained with Hoechst 33,342 (blue). In unaffected controls, ARMC4 and DNAH5 localized across the entire axonemal length. However, in the affected individuals, ARMC4 was undetectable, and DNAH5 failed to assemble in the distal ciliary compartment, confirming the recessive loss-of-function variants in *ARMC4*. Scale bars, 10 µm.

We analyzed the localization of ARMC4 and DNAH5 in PCD family members carrying loss-of-function variants of *ARMC4*. As expected, ARMC4 was absent from the ciliary axonemes, confirming the presence of pathogenic variants. Moreover, DNAH5 was localized only to the proximal axonemes in both siblings, consistent with what was previously reported (Lin et al., 2014) (Figures 4C,D).

## Discussion

Previous NGS-based studies in Turkish PCD cohorts have demonstrated considerable genetic heterogeneity, with pathogenic variants identified across multiple known PCD-related genes. Among these, *DNAH5* is frequently reported, whearas variability between studies likely reflects differences in cohort size and methodology (Demir et al., 2022; Emiralioğlu et al., 2020; Karakoç et al., 2025). The high rate of consanguinity observed in these populations increases the likelihood of homozygous pathogenic variants and highlights the importance of comprehensive genomic approaches such as whole-exome sequencing. However, a proportion of cases remain genetically unresolved, underscoring the complexity of PCD genetics.

Clinical findings across Turkish PCD cohorts frequently include laterality defects such as situs inversus and dextrocardia, reflecting the characteristic phenotype of the disease. Despite advances in genetic testing, a proportion of cases remain unresolved, highlighting the complexity of PCD genetics and the need for comprehensive diagnostic approaches such as WES. In this context, recent studies from Turkey have emphasized the practical value of IF analysis, particularly in settings where access to transmission electron microscopy (TEM) and high-speed video microscopy (HSVM) may be limited. IF provides rapid functional insight into ciliary protein defects and can support genetic findings when interpreted within the appropriate clinical framework (Karakoç et al., 2025).

**FIGURE 5:**
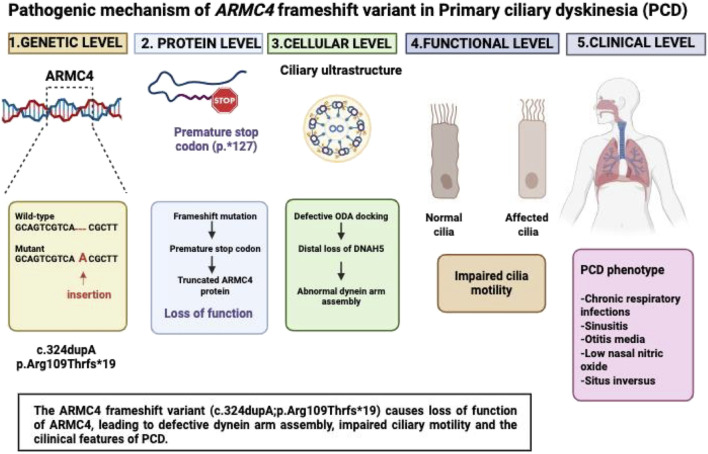
Schematic overview of the pathogenic mechanism of the ARMC4 frameshift variant. The identified ARMC4 variant results in a frameshift and premature stop codon, leading to a truncated protein and loss of function. This disrupts outer dynein arm (ODA) docking, resulting in abnormal localization of ciliary components such as DNAH5, impaired ciliary motility, and the clinical features of primary ciliary dyskinesia.

Consistent with these observations, our study demonstrates the added value of combining WES with IF analysis, whereby genetic findings are supported by functional evidence at the protein level, including absence of ARMC4 and altered DNAH5 localization (Hjeij et al., 2013). In the present study, we identified a novel frameshift variant in *ARMC4*, in exon three in a Turkish family with two affected siblings (Figure 5). The clinical features, including recurrent respiratory infections, sinusitis, otitis media, and low nasal nitric oxide levels, were consistent with PCD. The variant is predicted to introduce a premature stop codon, resulting in loss of protein function, which was supported by IF analysis demonstrating complete absence of ARMC4 in the ciliary axonemes in both affected individuals (FP1 and FP2).

ARMC4 is a key component of the ODA docking complex, required for proper attachment of dynein arms along the ciliary axoneme. Loss-of-function variants disrupt this process, leading to defective ciliary motility (Hjeij et al., 2013; Bolger et al., 2014; Li and Durbin, 2009; Picard, 2025; Poplin, 2018; Wang et al., 2010; James, 2011; Pike, 2017; Schwarz et al., 2014; Rentzsch et al., 2021; Ng and Henikoff, 2003; Landrum et al., 2018; Adzhubei et al., 2010; Choi et al., 2012; Shihab et al., 2014; Niroula and Vihinen, 2019; Hjeij et al., 2014; Richards et al., 2015; Lin et al., 2014; Demir et al., 2022; Emiralioğlu et al., 2020; Karakoç et al., 2025; Onoufriadis et al., 2013). Accordingly, our IF analysis demonstrated absence of *ARMC4* together with distal loss of DNAH5 and partial proximal retention, consistent with previously reported ARMC4-associated phenotypes and defective ODA docking. *ARMC4* variants reported in the literature include both missense and truncating mutations associated with defective ODA docking and characteristic IF patterns (Hjeij et al., 2013; Bolger et al., 2014; Li and Durbin, 2009; Picard, 2025; Poplin, 2018; Wang et al., 2010; James, 2011; Pike, 2017; Schwarz et al., 2014; Rentzsch et al., 2021; Ng and Henikoff, 2003; Landrum et al., 2018; Adzhubei et al., 2010; Choi et al., 2012; Shihab et al., 2014; Niroula and Vihinen, 2019; Hjeij et al., 2014; Richards et al., 2015; Lin et al., 2014; Demir et al., 2022; Emiralioğlu et al., 2020; Karakoç et al., 2025; Onoufriadis et al., 2013). A substantial proportion of these variants are predicted loss-of-function mutations, including nonsense and frameshift variants, and the variant identified in our study fits well within this mutation spectrum. From a clinical perspective, our findings highlight the value of integrating genetic and functional data, improving diagnostic accuracy and enabling more reliable interpretation of novel variants. Identifying novel variants in well-established PCD genes such as *ARMC4* contributes to expanding the mutational spectrum and supports more informed clinical decision-making and genetic counseling. Although the frequency of *ARMC4* mutations in Turkish patients with PCD is unknown due to the low number of NGS studies in the Turkish population, another *ARMC4* variation was previously reported in different consanguineous Turkish family members (Gileles-Hillel et al., 2020).

In conclusion, we identified a novel frameshift variant in *ARMC4 (*NM_001290020: p.Arg109Thrfs*19) in a Turkish family using WES and IF analyses. TEM could not be performed, representing a limitation of this study. Our findings contribute to the expanding mutational spectrum of ARMC4 and highlight the importance of integrating genetic and functional approaches in the diagnosis of PCD. These data may support improved variant interpretation and contribute to future developments in genetic diagnostics and patient management (Gileles-Hillel et al., 2020).

## Data Availability

The data presented in the study are deposited in the ClinVar repository (https://www.ncbi.nlm.nih.gov/clinvar/), accession number SCV004024742.
